# Influence of patient and tumor characteristics on therapy persistence with letrozole in postmenopausal women with advanced breast cancer: results of the prospective observational EvAluate-TM study

**DOI:** 10.1186/s12885-019-5806-y

**Published:** 2019-06-21

**Authors:** Markus Wallwiener, Naiba Nabieva, Manuel Feisst, Tanja Fehm, Johann de Waal, Mahdi Rezai, Bernd Baier, Gerold Baake, Hans-Christian Kolberg, Martin Guggenberger, Mathias Warm, Nadia Harbeck, Rachel Wuerstlein, Jörg-Uwe Deuker, Peter Dall, Barbara Richter, Grischa Wachsmann, Cosima Brucker, Jan Willem Siebers, Milos Popovic, Thomas Kuhn, Christopher Wolf, Hans-Walter Vollert, Georg-Peter Breitbach, Wolfgang Janni, Robert Landthaler, Andreas Kohls, Daniela Rezek, Thomas Noesselt, Gunnar Fischer, Stephan Henschen, Thomas Praetz, Volker Heyl, Thorsten Kühn, Thomas Krauss, Christoph Thomssen, Andre Hohn, Hans Tesch, Christoph Mundhenke, Alexander Hein, Claudia Rauh, Christian M. Bayer, Katja Schmidt, Erik Belleville, Sara Y. Brucker, Peyman Hadji, Matthias W. Beckmann, Diethelm Wallwiener, Sherko Kümmel, Andreas Hartkopf, Peter A. Fasching

**Affiliations:** 1 Department of Gynecology, University of Tübingen, Tübingen, Germany; 2 Department of Gynecology, University Hospital Heidelberg, Heidelberg, Germany; 3 Department of Gynecology, University Hospital Erlangen, Friedrich-Alexander University Erlangen-Nuremberg, Comprehensive Cancer Center Erlangen-EMN, Universitätsstrasse 21–23, 91054 Erlangen, Germany; 4 Institute of Medical Biometry and Informatics, University of Heidelberg, Heidelberg, Germany; 5 Department of Gynecology, Heinrich Heine University of Dusseldorf, Dusseldorf, Germany; 6 Department of Gynecology, Dachau Clinic, Dachau, Germany; 7 Luisen-Hospital Dusseldorf, Dusseldorf, Germany; 8 Oncological Medical Practice Pinneberg, Pinneberg, Germany; 9 Marien-Hospital Bottrop, Bottrop, Germany; 10 Tuttlingen Clinic, Tuttlingen, Germany; 11 Breast center, Department of Gynecology, University Hospital Cologne, Cologne, Germany; 12 Breast Center, Clinics of Cologne gGmbH Holweide, Cologne, Germany; 13 Department of Gynecology and Obstetrics, Breast Center and CCC Munich, University Hospital Munich, Munich, Germany; 14 Vinzenz-Hospital Hanover GmbH, Hanover, Germany; 15 Department of Gynecology, Lüneburg Clinic, Lüneburg, Germany; 16 Elbland Clinics, Meissen-Radebeul, Germany; 17 County hospital Böblingen, Böblingen, Germany; 18 Department of Gynecology and Obstetrics, Paracelsus Medical University, Nuremberg, Germany; 19 Department of Gynecology of the St. Josef’s Clinic Offenburg, Offenburg, Germany; 20 Department of Gynecology, Bayreuth clinic GmbH, CCC ER-EMN, Bayreuth, Germany; 21 Brustzentrum am Diakonie Klinikum Stuttgart, Stuttgart, Germany; 22 Medical Center ULM, Ulm, Germany; 23 Medical Campus Bodensee, Klinikum Friedrichshafen, Friedrichshafen, Germany; 24 Department of Gynecology, Neunkirchen Clinic, Neunkirchen, Germany; 25 Department of Gynecology, University Hospital Ulm, Ulm, Germany; 26 Gynecological Medical Practice of the County Hospital Krumbach, Krumbach, Germany; 27 Evangelic County Hospital Ludwigsfelde-Teltow, Ludwigsfelde-Teltow, Germany; 28 Marien-Hospital Wesel, Wesel, Germany; 29 Department of Gynecology of the Sana hospital Hameln, Hameln, Germany; 30 Mittweida Hospital gGmbH, Mittweida, Germany; 31 Johanniter Hospital Genthin Stendal gGmbH, Hansestadt Stendal, Germany; 32 Caritas-Hospital Bad Mergentheim, Bad Mergentheim, Germany; 33 Asklepios Paulinen Clinic Wiesbaden, Wiesbaden, Germany; 34 Department of Gynecology, Esslingen Clinics a.N, Esslingen, Germany; 35 Department of Gynecology Passau, Passau, Germany; 36 Department of Gynecology, Martin-Luther-University Halle-Wittenberg, Halle-Wittenberg, Germany; 37 Städtisches Krankenhaus Kiel GmbH, Kiel, Germany; 38 Oncology Bethanien Frankfurt, Frankfurt, Germany; 39 Department of Gynecology, University Hospital Schleswig-Holstein Campus Kiel, Kiel, Germany; 40 Novartis Pharma GmbH Nuremberg, Nuremberg, Germany; 41 Clin-Sol GmbH Würzburg, Würzburg, Germany; 42 Department of Gynecology, Nordwest Hospital, Frankfurt, Germany; 43 Breast center, Essen Mitte Clinics, Evang. Huyssens-Stiftung/Knappschaft GmbH, Essen, Germany

**Keywords:** Advanced/metastatic breast cancer, Palliative/metastatic treatment, Compliance, Persistence, Endocrine treatment/therapy, Aromatase inhibitor

## Abstract

**Background:**

Treatment of postmenopausal, hormone receptor-positive metastatic breast cancer (MBC) patients varies despite clear therapy guidelines, favoring endocrine treatment (ET). Aim of this study was to analyze persistence of palliative aromatase inhibitor (AI) monotherapy in MBC patients.

**Methods:**

EvAluate-TM is a prospective, multicenter, noninterventional study to evaluate treatment with letrozole in postmenopausal women with hormone receptor–positive breast cancer. To assess therapy persistence, defined as the time from therapy start to the end of the therapy (TTEOT), two pre-specified study visits took place after 6 and 12 months. Competing risk survival analyses were performed to identify patient and tumor characteristics that predict TTEOT.

**Results:**

Out of 200 patients, 66 patients terminated treatment prematurely, 26 (13%) of them due to causes other than disease progression. Persistence rate for reasons other than progression at 12 months was 77.7%. Persistence was lower in patients who reported any adverse event (AE) in the first 30 days of ET (89.5% with no AE and 56% with AE). Furthermore, patients had a lower persistence if they reported compliance problems in the past before letrozole treatment.

**Conclusions:**

Despite suffering from a life-threatening disease, AEs of an AI will result in a relevant number of treatment terminations that are not related to progression. Some subgroups of patients have very low persistence rates. Especially with regard to novel endocrine combination therapies, these data imply that some groups of patients will need special attention to guide them through the therapy process.

**Trial registration:**

Clinical Trials Number: CFEM345DDE19

**Electronic supplementary material:**

The online version of this article (10.1186/s12885-019-5806-y) contains supplementary material, which is available to authorized users.

## Background

Endocrine therapy (ET) is the recommended treatment in patients with hormone receptor-positive, advanced breast cancer. While, according to current guidelines, premenopausal women should receive tamoxifen as first-line therapy, aromatase inhibitors (AIs) or fulvestrant are preferred in postmenopausal metastatic breast cancer (MBC) patients [1, 2].

ET has recently become the focus of MBC treatment as novel combination therapies are being developed for hormone receptor-positive, advanced breast cancer patients to overcome endocrine resistance [3]. Adding the mTOR inhibitor everolimus to a therapy with the AI exemestane, for instance, improved progression-free survival (PFS) [4]. Furthermore, a combination of the cyclin-dependent kinase 4/6 (CDK 4/6) inhibitors palbociclib, ribociclib, or abemaciclib with ET has been investigated and has consistently shown a clinically relevant improvement in PFS [5–7].

To ensure ET efficacy, patient compliance and treatment persistence are needed in both the adjuvant and advanced setting. In the adjuvant setting, which has been investigated in several trials, compliance and persistence of therapy with tamoxifen or AIs in postmenopausal breast cancer patients decrease over the course of treatment [8–11], which, in turn, is associated with reduced disease-free survival (DFS) [12]. Some baseline patient and tumor characteristics such as age, socioeconomic factors, or tumor stage have been reported to have an influence [8–14]. In the advanced setting, in contrast, only few studies have analyzed patient compliance with ET, and thus not much is known about possible risk factors [15, 16].

Data concerning the persistence with regard to AI might not only be helpful for patients treated with a monotherapy but also for comparing persistence regarding endocrine combination therapies. Aim of this study was, therefore, to describe therapy persistence and to identify predictors for therapy persistence among those patient and tumor characteristics known at the start of treatment in a prospective, noninterventional study in patients receiving letrozole monotherapy in the metastatic setting. The hypothesis was that side effects and patient characteristics result in patients with different adherence rates.

## Methods

### Patients

EvAluate-TM is a prospective, multicenter, noninterventional, and observational study that evaluated treatment with the AI letrozole in postmenopausal hormone receptor-positive breast cancer patients in Germany [14, 17–19]. According to drug approval guidelines, patients received letrozole at 2.5 mg per day and were allowed to be on treatment up to 30 days before and required to start at maximum 30 days after signing informed consent. Follow-up took place for 12 months, while the last visit was allowed to be performed up to 3 months later. Besides, a minimum follow-up of 30 days was required for AE-analysis. Other inclusion or exclusion criteria were not defined. All patients provided written informed consent and all respective ethics committees approved the study.

### Data acquisition

Data on patient and tumor characteristics, including epidemiological characteristics, comorbidities, concomitant medication, as well as tumor stage and previous therapies, were entered into electronic case report forms. Patients were observed up to 15 months. At two prespecified study visits after 6 and 12 months from study inclusion, information about therapy compliance and whether the therapy had been stopped since the last visit was gathered from both patients and physicians. If the therapy had ended, the reason and date had to be documented. Furthermore, physicians and patients completed prespecified questionnaires about therapy compliance. Patients’ general health status and information on the perception of educational content provided on AI treatment was assessed.

### End points

The time from therapy start to the end of the therapy (TTEOT) was defined as therapy persistence. In the literature, terminology for describing compliance and persistence with therapy varies [12, 20, 21]. According to the current terminology, the treatment period is defined by the term persistence [20]. To simplify the discussion of study results, the term adherence is used as an overarching term for compliance and persistence in this analysis, which is in line with other studies [12, 22]. A patient was censored at the maximum observation time of 15 months according to the study plan or before, as the case may be, at the date of progression or death. The study aimed to evaluate the factors influencing the therapy decision of both the physician and the patient in standard care and assess patient management of therapy.

### Statistical methods

Patient population and patient characteristics were described with means, standard deviations (continuous variables), or absolute and relative frequencies (ordinal or dichotomous variables). As the trial was designed as an explorative study, all the *p*-values presented should be considered descriptive values.

Two competing risk survival analyses were performed to identify patient and tumor characteristics and items of the prespecified questionnaires that predict TTEOT. The competing risk was determined as the end of therapy due to disease progression and the end of therapy for reasons other than disease progression was determined as the event. The variables included in the analyses were selected due to their possible influence in parallel with a univariate analysis of the possible influencing factors (not reported). The first competing risk-survival analysis was based on patient and tumor characteristics as predictors of TTEOT, including the variables age at therapy begin (continuous), body mass index (continuous), ECOG (dichotomous, 0–1 and 2–4), number of different concomitant medications (integer), time from diagnosis to therapy (continuous), and adverse events (AE) in the first 30 days (dichotomous). The second competing risk-survival analysis considered the following items from the prespecified questionnaire (asked before beginning treatment with letrozole) as possible predictors of TTEOT: Do you sometimes forget to take your medicine? (yes/no); Do you take all your medicine always at the same time? (yes/no); Do you sometimes not take your medicine if you feel good? (yes/no); Do you not take your medicine at all if you feel worse due to illness? (yes/no); On how many days in the past 30 days did you not take/forget to take your medicine? (number of days); How satisfied were you with the information provided regarding endocrine treatment and its side effects? (from very satisfied to very unsatisfied on a scale of 1–5).

All statistical calculations were performed with the package *RiskRegression* of the statistics software R Version 3.4.1 and with the software SPSS Version 24.

## Results

Between 01/2008 and 12/2009 a total of 5045 patients were enrolled in the study, of whom 252 had advanced breast cancer. Of these, 52 women were excluded, out of which 28 patients were excluded because endocrine therapy started more than 30 days before signing the informed consent and 14 patients because treatment was started more than 30 days after the informed consent. In 4 patients the follow-up was too short (< 30 days) for side effect evaluation and in 6 patients data on disease progression or therapy compliance was missing. Thus, the current analysis consists of data of 200 MBC patients (Additional file 1: Figure S1).

### Patient characteristics

On average, the patients were 66.2 years old (SD = 11.3) and had a body mass index of 27.3 (SD = 5.4) kg/m^2^. Of the patients 60.0% had a pT2-T4 tumor and 56.0% had MBC at first diagnosis. Further patient and tumor characteristics are described in Table 1.

**Table 1:** Patient and tumor characteristics

Characteristics	mean or *N*	SD or %
Age (in years)	66.2	11.32
BMI (kg/m^2^)	27.3	5.40
ECOG at study entry		
0	81	40.5
1	93	46.5
2	20	10.0
3	5	2.5
4	1	0.5
pT at first diagnosis		
Unknown	13	6.5
pT0-pT1	67	33.5
pT2-pT4	120	60.0
pN at first diagnosis		
Unknown	42	21.0
pN0	58	29.0
pN1–3	100	50.0
cM at first diagnosis		
Unknown	4	2.0
cM0	84	42.0
cM1	112	56.0
Tumor grade at first diagnosis		
Unknown	6	3.0
G1	11	5.5
G2	137	68,5
G3	46	23.0
HER2/neu		
Unknown	26	13.0
Negative	147	73.5
Positive	27	13.5

The median observation time was 10.6 (SD = 3.9) months. A total of 66 (33.3%) therapy terminations were observed, of which 26 (13.0%) were for reasons other than disease progression or death.

The main nonprogression-related reason for premature treatment termination, which was reported at the time of treatment discontinuation, was side effects. Side effects were reported in 19 of the 26 events (9.5%). In one case (0.5%) therapy was discontinued due to the patient’s wish and in six cases (3.0%) for other reasons.

Persistence rate for patients with no progression during the observation time was 85.5% at month 12.

### Prediction of persistence

Descriptive statistics for the possible predictors for nonpersistence for reasons other than disease progression can be found in Table 2. The results of the two competing risk-survival analyses are shown in Table 3. In the first model the predictor “adverse events in the first 30 days” showed a significant *p*-value of *p* < 0.0001, indicating a possible influence on TTEOT. Hazard ratio (HR) was 8.24 (95% CI: 3.02–22.49) for patients with an AE compared to patients without. No other variable showed any significant influence on patients’ persistence in this model.

**Table 2:** Possible predictors for patients nonpersistent for reasons other than disease progression

Possible Predictors	Persistence		Non-persistence	
	Mean or *N*	SD or %	Mean or *N*	SD or %
Age (in years)	66.4	11.5	66.2	10.9
BMI (kg/m^2^)	27.6	5.7	27.6	4.8
Number of concomitant medications	2.1	3.2	1.7	2.3
ECOG				
0	56	41.8	13	50.0
1	62	46.3	10	38.5
2	11	8.2	3	11.5
3	4	3.0	0	0.0
4	1	0.7	0	0.0
Time from diagnosis to therapy (in years)	3.1	4.7	2.9	4.5
Adverse events within the first 30 days				
No	123	91.8	18	69.2
Yes	11	8.2	8	30.8
Do you sometimes forget to take your medicine?				
No	115	90.5	22	91.7
Yes	12	9.5	2	8.3
Do you take all your medicine always at the same time?				
No	11	8.7	2	8.3
Yes	115	91.3	22	91.7
Do you sometimes not take your medicine if you feel good?				
No	115	92.7	23	95.8
Yes	9	7.3	1	4.2
Do you not take your medicine at all if you feel worse due to illness?				
No	121	96.0	22	91.7
Yes	5	4.0	2	8.3
On how many days in the past 30 days did you not take/forget to take your medicine?				
0	109	93.2	20	87.0
1–10	8	6.8	3	13.0
How satisfied were you with the information provided regarding endocrine treatment and its side effects?				
Very satisfied	45	40.5	6	25.0
Satisfied	46	41.4	11	45.8
Neither satisfied nor unsatisfied	8	7.2	5	20.8
Unsatisfied	8	7.2	1	4.2
Very unsatisfied	2	1.8	1	4.2
Not applicable	2	1.8	0	0.0

**Table 3:** Prediction of time to end of treatment (TTOT) in patients not progressing under letrozole

	Possible predictors	Hazard Ratio	95% Confidence Interval		*p*-value
			Lower Bound	Upper Bound	
Competing Risk Regression Model 1	Age (in years)	1.02	0.97	1.08	0.373
	BMI (kg/m^2^)	1.02	0.94	1.11	0.563
	Number of concomitant medications	0.63	0.33	1.19	0.155
	ECOG	0.52	0.07	4,07	0.532
	Time from diagnosis to therapy (in years)	0.96	0.84	1.10	0.565
	Adverse events within the first 30 days	8.24	3.02	22.49	< 0.0001
Competing Risk Regression Model 2	Do you sometimes forget to take your medicine?	0.81	0.20	3.24	0.762
	Do you take all your medicine always at the same time?	1.15	0.23	5.68	0.864
	Do you sometimes not take your medicine if you feel good?	0.72	0.26	1.96	0.523
	Do you not take your medicine at all if you feel worse due to illness?	4.00	1.89	8.44	0.0003
	On how many days in the past 30 days did you not take/forget to take your medicine?	2.79	1.30	6.00	0.008
	How satisfied were you with the information provided regarding endocrine treatment and its side effects?	0.84	0.18	3.86	0.818

In the second model, the patients’ statements before therapy begin concerning treatment compliance in the past to any medication were tested. Their statement that they tend to “not taking medication if feeling ill” and the stated number of days patients were noncompliant with their concomitant medication over the previous 30 days showed a possible influence as predictors of TTEOT with *p*-values of *p* < 0.01. Patients who stated previous noncompliance if feeling ill had a higher risk for therapy termination than women who took their medication continuously (HR = 4.00; 95% CI: 1.89–8.44). Women who did not take their concomitant medication for at least 1 day also showed a higher risk for a lower persistence rate at 12 months with a HR of 2.79 (95% CI: 1.30–6.00).

**Fig. 1:**
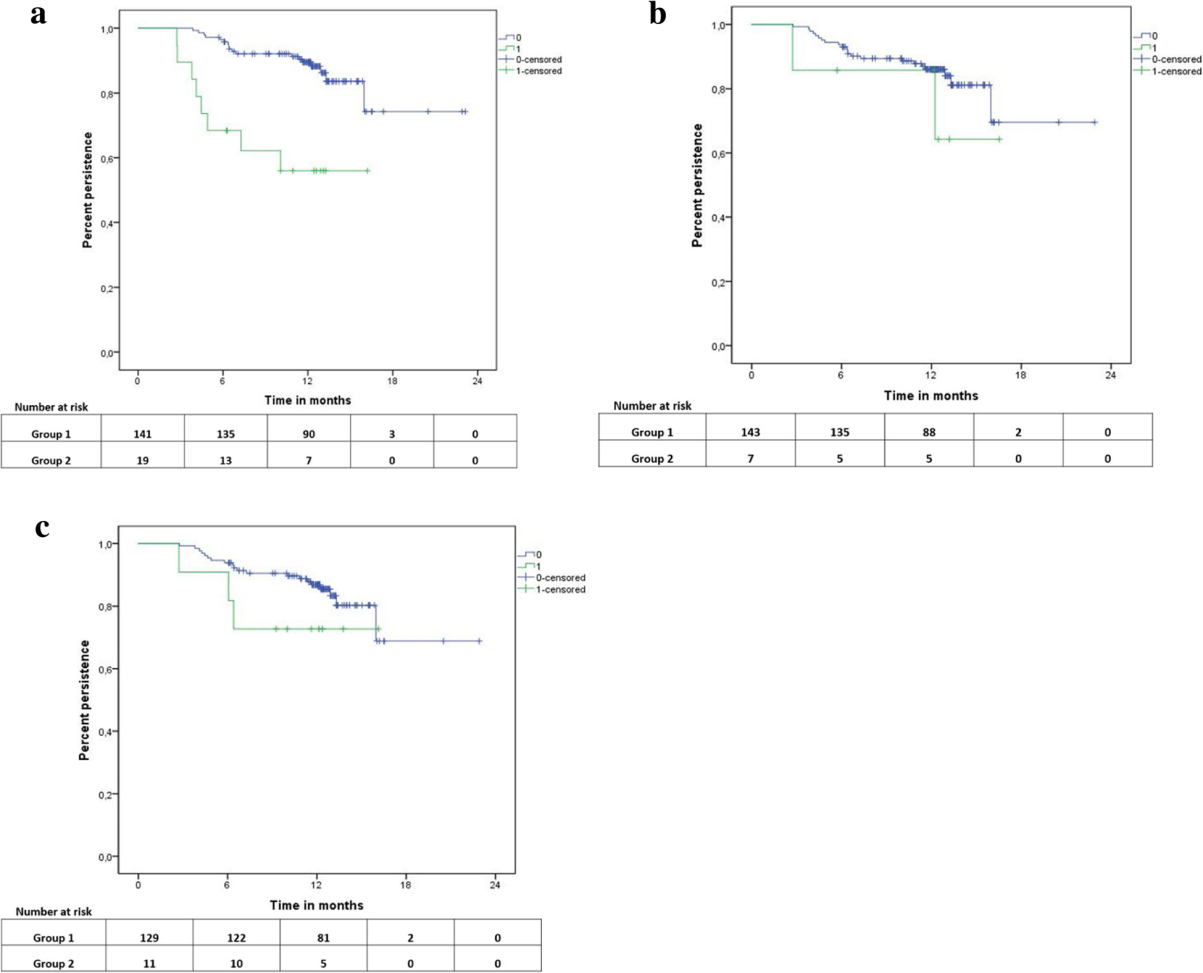
Kaplan–Meier curves for persistence for reasons other than disease progression: **a** dependent on adverse events within the first 30 days, **b** dependent on noncompliance due to illness, **c** dependent on noncompliance in the past 30 days. **a** Kaplan-Meier curves for at least one adverse event within the first 30 days after therapy start (0 = no adverse event; 1 = any adverse event). **b** Kaplan-Meier curves for the question „Do you not take your medicine at all if you feel worse due to illness?” (0 = False; 1 = True). **c**: Kaplan-Meier curves for the question „On how many days in the past 30 days did you not take/forget to take your medicine?” (0 = 0 days; 1 = 1–10 days)

Kaplan–Meier curves for possible predictors of persistence are shown in Fig. 1. Estimates for persistence rates at 12 months were 89.5 and 56.0% for patients without and with AEs, respectively. Persistence rates for patients, who stated they stopped therapy if feeling ill vs. those who did not state that, were 85.7 and 86.0%, respectively. With regard to women who did not take their concomitant medication for at least 1 day, the 12-month persistence rate was 72.7% compared with those who never missed taking their medication (86.9%).

## Discussion

After 12 months of observation, patients who were nonpersistent for reasons other than disease progression were still under AI treatment, with a persistence rate of 85.5%. In these patients, persistence was clearly compromised when AEs were reported within the first 30 days of treatment. Furthermore, statements about noncompliance in the past could also predict lower persistence.

ET with an AI not only reduces the recurrence rate of hormone receptor-positive breast cancer in the adjuvant setting [23], it also prolongs overall survival (OS) in those patients with advanced breast cancer [24]. As in the adjuvant setting adherence to AI therapy seems to have a direct influence on DFS [12], an important role in the treatment of MBC can be hypothesized.

In early breast cancer age [9, 13], comorbidities [9], prior chemotherapy or radiation [25, 26], tumor size [13], as well as socioeconomic factors [25] have been reported to have an influence on adherence to ET. In a Brazilian cohort of breast cancer patients, those who were diagnosed at a noncurable stage were less adherent to ET [25], while other contradictory analyses report that the stage at diagnosis seems to be associated with persistence, but not compliance [27]. Among MBC patients, there are only few analyses of adherence to ET [15, 16, 28]. An Italian investigator group observed among 285 postmenopausal MBC patients treated with exemestane that those who were married or had a university degree were less likely to not adhere to ET. Furthermore, older age at diagnosis, a higher number of comorbidities, as well as a lower receptivity toward therapy seemed to be associated with nonadherence. After 6 months of treatment, the adherence rate was 78% [15]. A recent analysis from Switzerland shows that out of 165 women who started palliative ET, a total of 12.8% did not persist (therapy termination or therapy change) with therapy due to side effects or for reasons other than disease progression. Those who were naïve to ET showed a higher persistence with palliative ET, while those with more metastatic lesions at diagnosis were less persistent [16]. In the FALCON study, 78.9% of the postmenopausal MBC patients receiving anastrozole discontinued treatment, among whom only 10.8% were for reasons other than disease progression. Of these treatment terminations, 4.7% were reported to be due mainly to adverse events. The median duration of actual exposure to anastrozole was 13.9 months [28].

Some of the aforementioned patient and tumor characteristics were also investigated in the present analysis. While none of the analyzed patient characteristics such as age, BMI, ECOG, or the number of concomitant medications had a significant influence on therapy persistence, AEs in the early treatment phase and patient noncompliance due to illness and forgetfulness correlate with nonpersistence. As mentioned before, literature on predictors of persistence with palliative ET is scarce, and thus it is difficult to bring these results in line with others. AI-induced side effects, which are described as the main reason for nonpersistence in the present work, represent one reason often given for noncompliance and an associated premature end of treatment [26, 29]. In the adjuvant setting, the COMPAS trial could demonstrate that compliance with AIs improves side effects, while noncompliant women were more likely to experience a deterioration of AE and might therefore discontinue treatment prematurely [30]. This might explain the vicious circle and why, in the current analysis, adverse events and noncompliance are in turn associated with a higher risk for nonpersistence.

For novel combination therapies, compliance rates are only available from prospectively randomized clinical trials. In the PALOMA-2 study with a median follow-up time of 23 months, an overall permanent discontinuation of study treatment as a result of AEs was reported in 43 patients (9.7%) in the palbociclib–letrozole group and in 13 patients (5.9%) in the placebo–letrozole group [5]. In the MONALEESA-2 study, at a median duration since randomization of 15.3 months, discontinuation due to AEs was reported in 87 patients (26.0%) in the ribociclib group and in 146 (43.7%) in the placebo group [7]. These figures are lower than those from our data. However, in clinical trials, compliance is generally thought to be higher for several reasons. Therefore, it will be important to observe real-world data that will capture this figure for patients on these novel combination therapies. However, it can be assumed that the rate will be lower than the 85.5% persistence rate that we reported for monotherapy.

A strength of this study is that, due to the nationwide patient recruitment, a broad MBC patient population is represented. Interesting is the high rate of MBC at first diagnosis, namely, 56.0%, which in the literature is reported to be only 5–10% [1], but is similar to the percentage in recent studies in that patient population [6]. Further trials report lower rates [4, 5, 7], which nevertheless, in comparison to epidemiologically known data, are high, so that the question arises as to whether there is a general increase in MBC at first diagnosis or whether this is based on a study selection bias. A weakness of our analysis is that, due to the small number of events (*n* = 26), the possible predictors for TTEOT were split into two competing risk regression models to achieve convergence and obtain robust results. Therefore, the results of the two models must be interpreted carefully by taking into account the separation of the predictors. A further weakness of the study is that data regarding compliance were collected by evaluating patient questionnaires and physicians’ assessments only at the time of enrollment and after 6 and 12 months. Since the median observation time in this study was only 10.6 months, but the median PFS of an ET with an AI is about 14.0 months according to the literature [28], it can be assumed that therapy persistence would even continue to decrease over the following months. As patients were not observed after the end of treatment, it also remains unknown whether women nonpersistent to letrozole treatment switched to another ET or were nonpersistent in general and discontinued therapy altogether. Importantly it has also to be noted that for our predictor women would have to be observed for 30 days with regard to the occurrence of side effects. Our findings can only be used for these women. For women who terminate the therapy before that time our findings are not applicable.

## Conclusion

The analysis suggests that the presence of AEs and statements about previous noncompliance can predict those women who will terminate palliative therapy with an AI. Despite suffering from a life-threatening disease and receiving a treatment that is generally considered as being well tolerated and thus the treatment of choice, AEs of an AI and a behavioral pattern related to noncompliance will result in a significant proportion of patients who prematurely terminate treatment. Therefore, further analyses are necessary to find predictive factors and identify MBC patients who are at risk for early treatment discontinuation and could benefit from supporting compliance programs. For example that up to 44% of women with side effects would terminate the therapy within 12 months of treatment compared to about 11% without side effects, makes this population a group of patients of interest who should be part of an intensified treatment management program. Furthermore, it should be investigated whether compliance and persistence patterns are the same with novel endocrine combination therapies.

## Additional file

Additional file 1: **Figure S1.** Patient flow chart (DOCX 93 kb)

## Data Availability

The datasets used and/or analysed during the current study are available from the corresponding author on reasonable request.

## Abbreviations

AE: Adverse event
AI: Aromatase inhibitor
CI: Confidence interval
DFS: Disease-free survival
ET: Endocrine therapy/treatment
HR: Hazard ratio
ICF: Informed consent form
MBC: Metastatic breast cancer
NIS: Non interventional study
OS: Overall survival
PFS: Progression-free survival
TTEOT: Time from therapy start to the end of the therapy
