# Supplementary figures and images for: Influence of patient and tumor characteristics on therapy persistence with letrozole in postmenopausal women with advanced breast cancer: results of the prospective observational EvAluate-TM study
Source: BMC Cancer. 2019 Jun 21;19:611. doi: 10.1186/s12885-019-5806-y (PMC6588890; doi:10.1186/s12885-019-5806-y)

Additional file 1: Figure S1: Patient flow chart


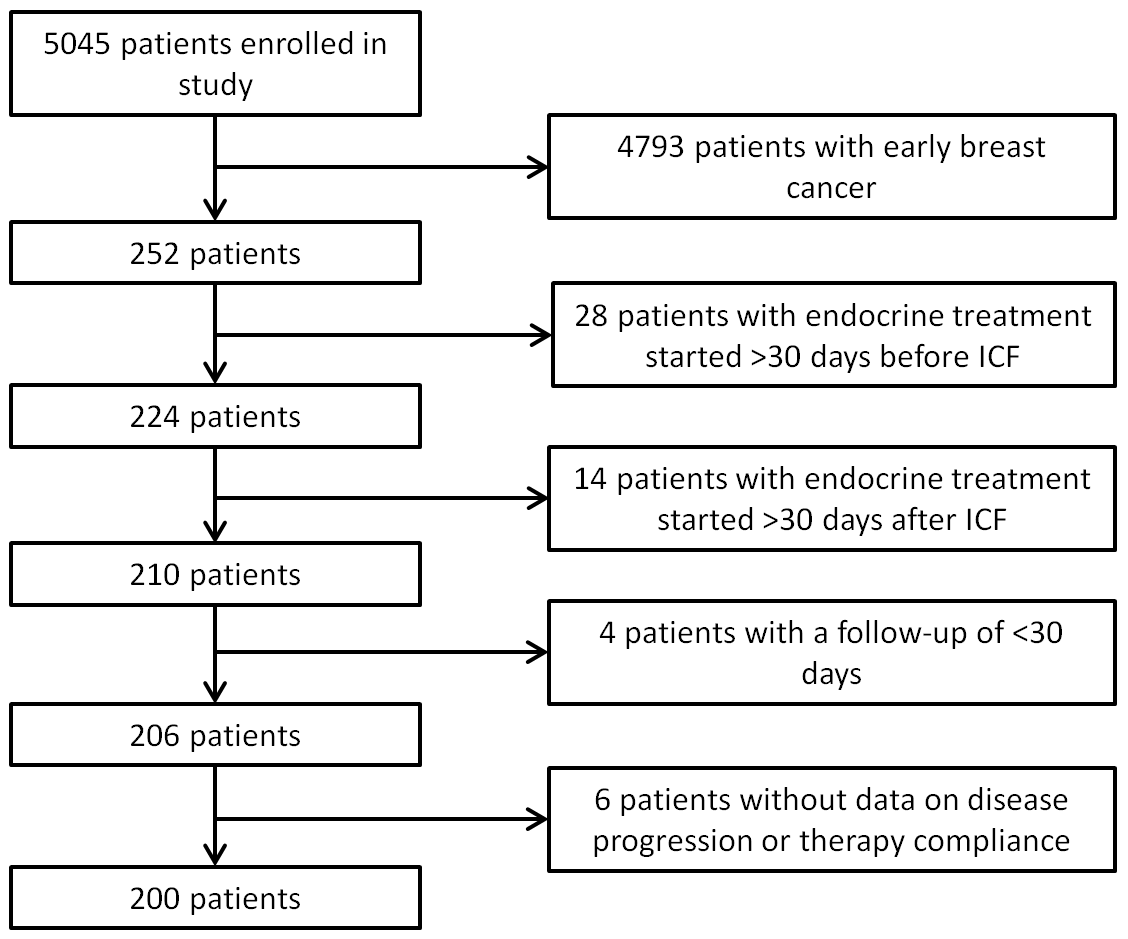

Supplement: Supplementary file 1 — Figure S1. Patient flow chart (DOCX 93 kb) [file 12885_2019_5806_MOESM1_ESM.docx]
